# Outcomes of Emergency Transcatheter Aortic Valve Replacement

**DOI:** 10.1155/2019/7598581

**Published:** 2019-11-03

**Authors:** Hans Huang, Christopher P. Kovach, Sean Bell, Mark Reisman, Gabriel Aldea, James M. McCabe, Danny Dvir, Creighton Don

**Affiliations:** ^1^Division of Cardiology, Department of Medicine, University of Washington, Seattle, WA, USA; ^2^Division of Pulmonary, Critical Care and Sleep Medicine, Department of Medicine, University of Washington, WA, Seattle, USA; ^3^Department of Medicine, University of Washington, Seattle, WA, USA; ^4^Division of Cardiothoracic Surgery, Department of Surgery, University of Washington, Seattle, WA, USA

## Abstract

**Objective:**

To identify outcomes of patients undergoing emergency transcatheter aortic valve replacement (TAVR) and determine predictors of in-hospital mortality.

**Background:**

Emergency TAVR has emerged as a viable treatment strategy for patients with decompensated severe aortic stenosis and/or regurgitation; however, data on patients undergoing emergency TAVR are limited.

**Methods:**

All emergency TAVR procedures were identified from a single tertiary academic center between January 2015 and August 2018.

**Results:**

31 patients underwent emergency TAVR due to cardiogenic shock (26 patients), electrical instability with incessant ventricular tachycardia (2 patients), severe refractory angina (2 patients), and decompensated heart failure with hypoxemic respiratory failure requiring mechanical ventilation (1 patient). Mechanical circulatory support (MCS) was used in 16 (51.6%). MCS initiation occurred immediately prior to TAVR in 10 patients and placed post-TAVR in 6 patients. 6 patients died before hospital discharge (in-hospital mortality 19.4%). 1-year and 2-year survival rates were 61.0% and 55.9%, respectively. Univariate predictors of in-hospital mortality were preprocedural pulmonary artery pulsatility index (PAPi) ≤1.8 (66.7% vs. 20.0%, *p*=0.01), intraprocedural cardiopulmonary resuscitation (CPR) (83.3% vs 4.0%, *p* ≤ 0.001), acute kidney injury post-TAVR (80.0% vs. 4.2%, *p* ≤ 0.001), initiation of dialysis post-TAVR (60.0% vs. 4.2%, *p* ≤ 0.001), and MCS initiation post-TAVR (50.0% vs. 12.0%, *p*=0.03). MCS initiation before TAVR was associated with improved survival compared with post-TAVR initiation.

**Conclusion:**

Emergency TAVR in extreme risk patients with acute decompensated heart failure or cardiogenic shock secondary to severe aortic valve disease is associated with high in-hospital mortality rates. Careful patient selection taking into account right heart function, assessed by PAPi, and early utilization of MCS may improve survival following emergency TAVR.

## 1. Introduction

Acute decompensated heart failure or cardiogenic shock due to severe aortic stenosis (AS) portends a dismal diagnosis, and operative risk in these patients is exceedingly high [1, 2]. Catheter-based procedures such as emergency balloon aortic valvuloplasty and emergency transcatheter aortic valve replacement (TAVR) have provided alternative treatment options in these unstable, high-risk patients. Balloon aortic valvuloplasty has been proposed as a therapy to allow a bridge to treatment or to decision, but carries risks for strokes and vascular complications and may not be sufficient to stabilize a decompensated patient [3]. TAVR has been successfully established as the treatment for patients with severe AS who are at prohibitive or high risk for surgical aortic valve replacement [4, 5] and is emerging as a viable treatment strategy in the urgent/emergency setting for hemodynamically unstable patients [6]. The largest study to date, utilizing the Society of Thoracic Surgeons/American College of Cardiology Transcatheter Valve (STS/ACC TVT) Registry, reported favorable 30-day and 1-year outcomes of 3,952 patients when combining urgent and emergency TAVR procedures; however, emergency TAVR cases represented only 0.2% of the study population, and salvage cases were excluded [6]. Thus, results from this registry-based study are not reflective of emergency TAVR outcomes. Further studies are needed to identify the role of emergency TAVR and identify patients who may benefit from this intervention.

In this study, we performed a single-center retrospective case series to determine survival rates and in-hospital outcomes in unstable patients undergoing emergency TAVR and to identify risk factors associated with outcomes among these patients.

## 2. Methods

### 2.1. Study Population and Definitions

Emergency TAVR procedures were identified from chart review of all TAVR cases performed at the University of Washington Medical Center between January 2015 and August 2018. The study was approved by the Institutional Review Board at the University of Washington.

In accordance with the TVT Registry [7], we defined an emergency condition as one in which an immediate operative aortic valve intervention was required due to “ongoing, refractory (difficult, complicated, and/or unmanageable), unrelenting cardiac compromise, with or without hemodynamic instability, and not responsive to any form of medical therapy except cardiac surgery.” Examples include patients in cardiogenic shock requiring catecholamine therapy or mechanical circulatory support (MCS), electrical instability (e.g., incessant ventricular tachycardia), or severe refractory respiratory failure due to congestive heart failure requiring mechanical ventilation. Patients who were actively receiving cardiopulmonary resuscitation (CPR) at the time of TAVR deployment were also included in this study; these cases have been previously defined by the TVT Registry as a salvage TAVR [7]. We considered a patient to be in cardiogenic shock if he or she met the following criteria: systolic blood pressure of less than 90 mmHg for longer than 30 minutes or the use of catecholamine therapy or mechanical circulatory support to maintain a systolic pressure of at least 90 mmHg, clinical signs of pulmonary congestion, and signs of impaired organ perfusion (e.g., altered mental status, decreased urine output, acute kidney injury, and elevated lactate) [8–11]. Hemodynamic assessment of the patients in the study cohort generally occurred after initiation of inotropic or mechanical circulatory support, and thus cardiac index measurements were not used to define cardiogenic shock.

### 2.2. Data Collection and Outcomes

Patient demographic data, echocardiographic variables, cardiac catherization hemodynamic data, and clinical outcomes were obtained from the electronic medical record via chart review. Echocardiographic images were reviewed when necessary to obtain specific variables. Cardiac catheterization hemodynamic variables were obtained by right heart catheterization performed immediately prior to TAVR or from bedside pulmonary artery catheter during the 24 hours preceding TAVR.

Outcomes were determined by chart review. The primary endpoint was all-cause mortality. Secondary in-hospital outcomes included intraprocedure mortality, procedural success, postprocedure aortic regurgitation, stroke, major bleed, major vascular complication, myocardial infarction (MI), new permanent pacemaker (PPM), KDIGO stage III acute kidney injury (AKI), initiation of dialysis post-TAVR, and hospital length of stay. All in-hospital outcomes were defined using standardized consensus-derived criteria from the Valve Academic Research Consortium II [12].

### 2.3. Statistics

Numbers were given as number (%), mean, and standard deviations for quantification of normally distributed variables or median and interquartile ranges for nonnormally distributed variables in the tables. For comparison of normally distributed variables, Student's *t*-test was used. For comparison of patient characteristics, chi-square test was applied. Cumulative mortality was estimated by the Kaplan–Meier method and compared by the log-rank test. Regression analysis by using pseudoobservations was used to compare survival functions and compensate for incomplete right-censored time-to-event data. Univariate Cox regressions were performed including all variables potentially associated with mortality. A multivariate regression analysis was attempted but unable to be performed due to the low number of events and significant collinearity between variables. Data were analyzed using STATA 15 (STATA Corp, LLC, College Station, TX) and GraphPad Prism 7.0 (GraphPad Software, Inc., La Jolla, CA) to create figures. *p*-values <0.05 were considered to be statistically significant.

## 3. Results

### 3.1. Baseline Characteristics and Echocardiographic and Hemodynamic Variables

**Figure 1:**
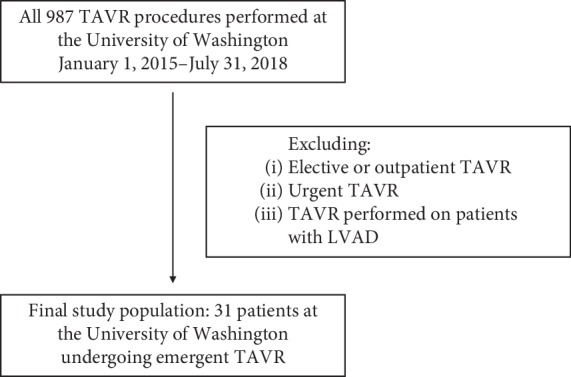
Flow chart showing selection of study population. LVAD: left ventricular assist device; TAVR: transcatheter aortic valve replacement.

Of the 987 patients who underwent TAVR at the University of Washington from January 2015 to August 2018, we identified 31 patients undergoing emergency TAVR (Figure 1). Median follow-up time was 311 days. Demographic data, comorbidities, and preprocedural characteristics are presented in Table 1. The mean age was 73.1 ± 13.9 years and 74.2% were men. Emergency TAVR was performed due to refractory cardiogenic shock (26 patients); electrical instability with incessant ventricular tachycardia (2 patients); severe unrelenting angina despite medical therapy (2 patients); and decompensated heart failure with refractory hypoxemic respiratory failure requiring mechanical ventilation (1 patient).

Baseline echocardiographic variables are listed in Table 2. 24 patients (77.4%) had a calculated aortic valve area of ≤1.0 cm^2^. The majority of patients had findings consistent with low-flow, low-gradient aortic stenosis: severely reduced left ventricular systolic function with an ejection fraction of <35% (19 patients, 61.3%), indexed stroke volume ≤35 ml/m^2^ (22 patients, 71%), and a mean valve gradient <40 mmHg (24 patients, 77.4%). Invasive hemodynamic assessment pre-TAVR revealed a mean pulmonary artery pressure of 35.4 ± 7.3 mmHg, pulmonary capillary wedge pressure of 24.9 ± 7.9 mmHg, cardiac index of 2.6 ± 1.0 l/min/m^2^, and cardiac power index of 0.41 ± 0.17 W/m^2^ (Table 3). 9 patients did not have a hemodynamic assessment performed prior to TAVR.

### 3.2. Procedural Characteristics

Procedural characteristics are shown in Table 4. 27 patients underwent emergency TAVR for primary AS, 3 for primary AI, and 1 patient had mixed AS/AI. Use of MCS pre- or post-TAVR occurred in 16 patients (pre-TAVR: 10 patients; post-TAVR: 6 patients). Intra-aortic balloon pump (IABP) was the most common form of MCS pre-TAVR, while Impella support was most common post-TAVR. 25 patients (80.6%) received a balloon-expanding valve, and 5 patients (16.1%) received a self-expanding valve. Immediate procedural success (defined as correct positioning of transcatheter valve with intended valve performance, and no intraprocedural death) was achieved in 29 patients (Table 5). One device failure was due to embolization of the valve from a ruptured delivery balloon and the other was due to intraprocedural death.

### 3.3. Outcomes

**Figure 2:**
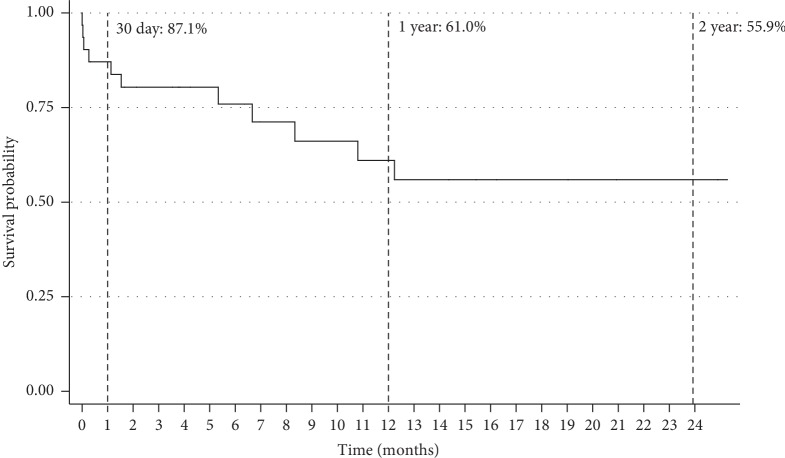
Kaplan–Meier curve for mortality following emergency transcatheter aortic valve replacement.

**Figure 3:**
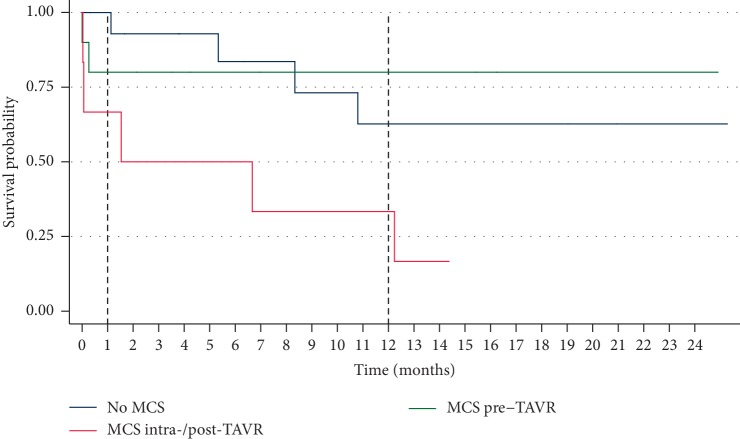
Kaplan–Meier curve for mortality following emergency transcatheter aortic valve replacement (TAVR) without mechanical circulatory support (MCS), MCS pre-TAVR, and MCS intra-/post-TAVR.

**Figure 4:**
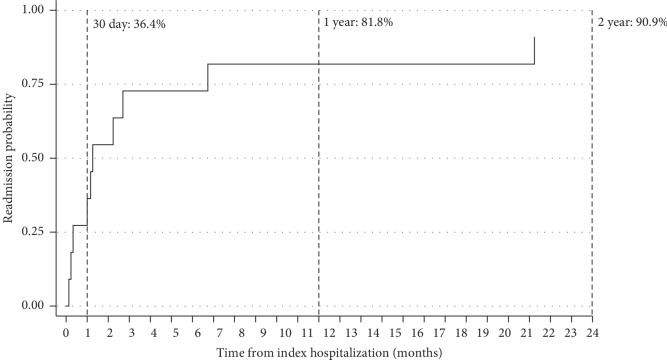
Kaplan–Meier curve for readmission after emergency transcatheter aortic valve replacement.

6 patients (19%) died in-hospital, and 13 (42%) died over total follow-up. 7 deaths (54%) were from cardiovascular causes and 6 (46%) from noncardiovascular causes. During the postprocedure hospital course, 3 patients suffered a stroke, 2 patients experienced a major bleeding event, 1 suffered a myocardial infarction, and 2 required pacemaker placement for bradyarrhythmia. 5 patients developed AKI and 4 required initiation of dialysis. Other in-hospital outcomes are listed in Table 5. Similar outcomes were observed when excluding patients with primary aortic regurgitation undergoing emergency TAVR (Supplemental [Supplementary-material supplementary-material-1]). Overall Kaplan–Meier estimates of survival at 30 days, one year, and two years were 87.1%, 61.0%, and 55.9%, respectively (Figure 2). Further subgroup analysis revealed that patients in whom MCS was instituted prior to TAVR had similar survival rates compared to patients who did not receive MCS at 30 days (80.0% vs. 100%; *p*=0.12), 1 year (80.0% vs. 62.7%; *p*=0.47), and 2 years (80.0% vs. 62.7%; *p*=0.51). Conversely, patients who required rescue MCS intraprocedurally had lower overall survival at 30 days (66.7%) and 1 year (33.3%) that was statistically significant at completion of follow-up compared with those who received MCS pre-TAVR (*p* < 0.01) or those in whom MCS was not utilized (*p*=0.04) (Figure 3). Comparison of baseline characteristics of patients who received MCS pre-TAVR compared with MCS placed intraprocedurally is shown in Supplementary [Supplementary-material supplementary-material-1]. Overall readmission rates at 30 days, 1 year, and 2 years were 36.4%, 81.8%, and 90.9%, respectively (Figure 4).

The 6 patients who died in-hospital differed significantly from patients who survived to hospital discharge with respect to preprocedural pulmonary artery pulsatility index (PAPi) ≤1.8 (66.7% vs. 20.0%, *p*=0.01), intraprocedural cardiopulmonary resuscitation (CPR) (83.3% vs. 4.0%, *p* ≤ 0.001), acute kidney injury post-TAVR (80.0% vs. 4.2%, *p* ≤ 0.001), initiation of dialysis post-TAVR (60.0% vs. 4.2%, *p* ≤ 0.001), and MCS initiation post-TAVR (50.0% vs. 12.0%, *p*=0.03) (Table 6).

## 4. Discussion

Our study shows that emergency TAVR in patients presenting with cardiogenic shock or extremis due to severe aortic valvular disease is a feasible strategy with acceptable mortality rates. Our cohort treated with emergency TAVR was critically ill and at extremely high risk with a majority of patients in cardiogenic shock on inotropic support and large proportion of patients requiring MCS and/or mechanical ventilation at the time of TAVR. Despite the critical condition of these patients, we report a procedural success rate of 93.5% with a single intraprocedural death (3.3%) and an acceptable 30-day and 1-year survival probability of 87% and 61%, respectively. The in-hospital mortality rate of emergency TAVR was 19.4% with five of six patient deaths due to cardiovascular causes.

Not surprisingly, the 30-day mortality of our emergency cohort exceeded that of elective TAVR in high-risk nonsurgical candidates, which amounted to 5.0% in the Partner B study [4]. However, with a longer follow-up, mortality seen in Partner B is comparable to our results with 1- and 2-year all-cause mortality rate of 30.7% and 43.3%, respectively. More recent studies have evaluated outcomes of TAVR in the setting of aortic decompensation and shock. Kolte et al. performed the largest study of urgent/emergency TAVR outcomes using the STS/ACC TVT Registry [6]. The primary analysis of this study combined both urgent and emergency TAVRs for a total of 3,952 procedures (64 defined as emergency). Of note, only 2.5% of patients were in cardiogenic shock within 24 hours of TAVR and only 1.2% of patients had a mechanical assist device in place at the start of the procedure. The 30-day and 1-year mortality rates were 8.7% and 29.1%, respectively, for urgent TAVR, whereas the 30-day and 1-year mortality rates for emergency TAVR were 12.5% and 32.2%, respectively. These results are comparable to our mortality rate at 30-days of 12.9% and at 1-year of 39%. The STS/ACC TVT study excluded salvage TAVR and patients who had a cardiac arrest within 24 hours prior to procedure, so it is encouraging that our survival rates were similar, even with the inclusion of these patients. Smaller studies evaluating outcomes in emergency TAVR have reported higher 30-day mortality rates [13, 14]. Frerker et al. performed a single-center study evaluating outcomes of 27 patients undergoing emergency TAVR for severe aortic stenosis in the setting of cardiogenic shock. They reported a high acute device success rate of 88.9% and a 30-day and 1-year mortality of 33.3% and 40.7%, respectively [13].

In our study, six patients had MCS placed after valve deployment during the initial TAVR procedure. In four of the six patients, MCS was placed immediately as a “bail-out” strategy in the setting of cardiac arrest and CPR during the procedure; all four of these patients died during the follow-up period from refractory cardiogenic shock. Unsurprisingly, CPR during TAVR was associated with mortality, and emergency percutaneous mechanical support could not salvage these patients. The high short-term mortality for emergency “bail out” use of MCS during TAVR in extremely high-risk populations has also been reported [15]. Of the 10 patients in our cohort who had MCS pre-TAVR, only 2 died in-hospital, and their long-term survival mirrored that of our larger cohort. While we cannot exclude that patient selection biased these results, it is conceivable that early stabilization may allow patients to better tolerate the hemodynamic perturbations associated with TAVR. This finding highlights the importance of early identification of patients with very poor cardiac and functional reserve who should be stabilized pre-TAVR, potentially benefiting from upfront use of MCS. Our results suggest patients with impaired right ventricular function, as evidenced by a low PAPi, are at higher risk of in-hospital mortality with emergency TAVR. These patients may represent a subset who would derive benefit from early upfront mechanical support. Larger registry data evaluating outcomes of emergency TAVR are needed to identify independent predictors of mortality for this extremely high-risk group of patients and help inform selective use of pre-TAVR MCS support.

Our study is limited by its small sample size, single-center design, and retrospective nature. We only evaluated patients who underwent a procedure, and thus there is an inherent potential for case selection bias. This study did not identify the larger cohort of patients in our center who were not considered candidates for TAVR, and therefore, our findings may not be applicable to all patients with cardiogenic shock and aortic valve disease, given the possibility of unmeasured confounding variables. Cardiogenic shock was defined post hoc primarily by clinical criteria (e.g., need for inotropic or mechanical circulatory support and end-organ injury) in this cohort. The mean cardiac index and mean arterial pressures documented by right heart catheterization were above those typically associated with shock; however, these hemodynamic assessments were generally performed after patients had already been receiving support and represent an artifact of the study's retrospective design. Among patients our interdisciplinary heart team agreed to treat, despite extreme risk and the fact these patients have been excluded from previous studies, we show very favorable outcomes. Due to the low number of study patients, secondary analysis comparing subgroups limits the robustness of these data and the confidence that they are not simply caused by chance. We acknowledge all the limitations of a single-center observational trial with relatively small numbers. Nonetheless, the number of patients included in this study represents one of the largest single-center experiences of patients undergoing emergency TAVR to date.

## 5. Conclusions

Emergency TAVR can be a viable option for decompensated, very unstable patients with severe aortic valve disease with an in-hospital mortality rate of 19.4% and long-term outcomes similar to studies of extreme risk patients treated electively. Careful patient selection, taking into account baseline hemodynamic parameters, such as PAPi, and early utilization of MCS may improve survival following emergency TAVR. Prospective validation of our findings within a large cohort of patients is warranted.

## Figures and Tables

**Table 1:** Baseline demographic and clinical characteristics of patients undergoing emergency transcatheter aortic valve replacement.

*Demographics*	
Age (years), mean (SD)	73.1 (13.9)
Male gender, *n* (%)	23 (74.2)
BSA (kg/m^2^), mean (SD)	1.94 (0.24)
Race	
White, *n* (%)	22 (71.0)
Black, *n* (%)	1 (3.2)
Hispanic, *n* (%)	2 (6.5)
Asian, *n* (%)	3 (9.7)
Other/unknown, *n* (%)	3 (9.7)

*History and Risk Factors*	
Current/recent smoker, *n* (%)	6 (19.4)
Hypertension, *n* (%)	25 (80.6)
Diabetes mellitus, *n* (%)	12 (38.7)
Peripheral artery disease, *n* (%)	4 (12.9)
Atrial fibrillation, *n* (%)	15 (51.6)
Coronary artery disease, *n* (%)	21 (67.7)
Chronic kidney disease (GFR <60 ml/min; not on HD), *n* (%)	12 (38.7)
Chronic kidney disease on HD, *n* (%)	2 (6.5)
COPD, *n* (%)	3 (9.7)
Severe COPD, *n* (%)	1 (3.2)
Prior PCI, *n* (%)	10 (32.3)
Prior CABG, *n* (%)	6 (19.4)
Prior sternotomy, *n* (%)	7 (22.6)
Prior SAVR, *n* (%)	5 (16.1)
Prior stroke, *n* (%)	1 (3.2)
Immunocompromise, *n* (%)	2 (6.5)
Known history of CHF, *n* (%)	26 (83.9)

*Preprocedure Status*	
Prior MI, *n* (%)	22 (71.0)
Cardiogenic shock within 24 hours, *n* (%)	26 (83.9)
ACS at hospital presentation, *n* (%)	15 (48.4)
ACS at time of TAVR, *n* (%)	12 (38.7)
Heart rate (beats/min), mean (SD)	92.7 (20.1)
BNP (pg/mL), mean (SD)	2217.0 (1759.2)
Creatinine (mg/dL), mean (SD)	1.56 (1.23)
Intubated, *n* (%)	12 (38.7)

BSA: body surface area; GFR: glomerular filtration rate; HD: hemodialysis; COPD: chronic obstructive pulmonary disease; PCI: percutaneous coronary intervention; CABG: coronary artery bypass grafting; SAVR: surgical aortic valve replacement; CHF: congestive heart failure; MI: myocardial infarction; ACS: acute coronary syndrome; TAVR: transcatheter aortic valve replacement; BNP: B-type natriuretic peptide.

**Table 2:** Baseline echocardiographic variables.

Aortic valve area (cm^2^), mean (SD)	0.96 (0.69)
</ = 1.0 cm^2^, *n* (%)	24 (77.4)

Mean valve gradient (mmHg), mean (SD)	30.9 (14.8)
>/ = 40 mmHg, *n* (%)	7 (22.6)

Moderate/severe AR, *n* (%)	9 (29.0)
Moderate/ severe MR, *n* (%)	8 (25.8)

LVEF (%), mean (SD)	31.8 (15.3)
LVEF<35%, *n* (%)	19 (61.3)

Stroke volume index (ml/m^2^), mean (SD)	31.4 (12.5)
</ = 35 ml/m^2^, *n* (%)	22 (71.0)

Estimated PASP (mmHg), mean (SD)	47.0 (8.0)

AR: aortic regurgitation; MR: mitral regurgitation; LVEF: left ventricular ejection fraction; PASP: pulmonary artery systolic pressure.

**Table 3:** Pretranscatheter aortic valve replacement hemodynamics.

Right atrial pressure (mmHg), mean (SD)	12.2 ± 5.4

Pulmonary artery pressure	
Systolic (mmHg), mean (SD)	50.7 ± 11.0
Diastolic (mmHg), mean (SD)	26.9 ± 6.5
Mean (mmHg), mean (SD)	35.4 ± 7.3

Pulmonary capillary wedge pressure or LVEDP (mmHg), mean (SD)	24.9 ± 7.9

Mean arterial pressure (mmHg), mean (SD)	70.3 ± 10.7

Cardiac output (l/min), mean (SD)	5.1 ± 1.9

Cardiac index (l/min/m^2^), mean (SD)	2.6 ± 1.0

Cardiac power index (W/m^2^), mean (SD)	0.41 ± 0.17
Cardiac power index on MCS pre-TAVR (W/m^2^), mean (SD)	0.38 ± 0.12

LVEDP: left ventricular end diastolic pressure; MCS: mechanical circulatory support; TAVR: transcatheter aortic valve replacement.

**Table 4:** Procedural characteristics.

Procedural indication	
Primary aortic stenosis, *n* (%)	27 (87.0)
Primary aortic insufficiency, *n* (%)	3 (9.7)
Mixed aortic stenosis/insufficiency, *n* (%)	1 (3.2)

Device used	
Balloon-expandable valve, *n* (%)	25 (80.6)
Self-expanding valve, *n* (%)	5 (16.1)

Contrast volume (ml), mean (SD)	97.2 (64.8)

PCI at time of TAVR, *n* (%)	6 (19.4)

Intraprocedural CPR, *n* (%)	6 (19.4)

MCS in place at start of TAVR	10 (32.3)
IABP, *n* (%)	7 (22.6)
Impella, *n* (%)	3 (9.7)
TandemHeart, *n* (%)	1 (3.2)

MCS placed after TAVR deployment	6 (19.4)
IABP, *n* (%)	2 (6.5)
Impella, *n* (%)	5 (16.1)
TandemHeart, *n* (%)	0 (0.0)

Day of procedure APACHE II score, mean (SD)	13.3 (6.3)

PCI: percutaneous coronary intervention; TAVR: transcatheter aortic valve replacement; CPR: cardiopulmonary resuscitation; MCS: mechanical circulatory support; IABP: intra-aortic balloon pump; APACHE: acute physiology and chronic health evaluation.

**Table 5:** Outcomes.

In-hospital outcomes	
Intraprocedural mortality, *n* (%)	1 (3.3)
In-hospital mortality, *n* (%)	6 (19.4)
Procedural success, *n* (%)	29 (93.5)
AR postprocedure-none/mild, *n* (%)	30 (100.0)
AR postprocedure-moderate/severe, *n* (%)	0 (0.0)
Stroke, *n* (%)	3 (10.0)
Major bleed, *n* (%)	2 (6.7)
Major vascular complication, *n* (%)	5 (16.7)
Myocardial infarction, *n* (%)	1 (3.3)
New PPM, *n* (%)	2 (6.7)
Acute kidney injury stage III, *n* (%)	5 (16.7)
Initiation of dialysis post-TAVR, *n* (%)	4 (13.3)
LOS (days), mean (SD)	12.4 (7.8)

All-cause mortality	
In-hospital, *n* (%)	6 (19.4)
Total follow-up, *n* (%)	13 (41.9)

Cause of death	
Cardiovascular mortality, *n* (%)	7 (53.8)
Noncardiovascular mortality, *n* (%)	6 (46.2)

AR: aortic regurgitation; PPM: permanent pacemaker; TAVR: transcatheter aortic valve replacement; LOS: length of stay.

**Table 6:** Comparison of patients undergoing emergency transcatheter aortic valve replacement according to survival status at hospital discharge.

	Survivor (*n* = 25)	Nonsurvivor (*n* = 6)	*p*-value
Preprocedure history of atrial fibrillation, *n* (%)	11 (44.0)	5 (83.3)	0.08
Heart rate pre-TAVR (BPM), mean (SD)	94.6 (20.1)	84.6 (19.7)	0.1
Estimated PASP (mmHg), mean (SD)	48.3 (7.5)	40.1 (8.3)	0.03
Pulmonary artery pulsatility index, mean (SD)	2.58 (1.57)	1.32 (0.41)	0.07
Pulmonary artery pulsatility index ≤1.8, *n* (%)	5 (20.0)	4 (66.7)	0.01

MCS placement post-TAVR deployment, *n* (%)	3 (12.0)	3 (50.0)	0.03
Intraprocedural CPR, *n* (%)	1 (4.0)	5 (83.3)	≤0.001

Acute kidney injury post-TAVR, *n* (%)	1 (4.2)	4 (66.7)	≤0.001
Initiation of dialysis post-TAVR, *n* (%)	1 (4.2)	3 (60.0)	≤0.001

Postprocedure stroke, *n* (%)	2 (8.3)	1 (16.7)	0.76

TAVR: transcatheter aortic valve replacement; BPM: beats per minute; PASP: pulmonary artery systolic pressure; MCS: mechanical circulatory support; CPR: cardiopulmonary resuscitation.

## Data Availability

The data used to support the findings of this study are included within the article and supplementary tables.

## Supplementary Materials

Supplementary Materials Two additional tables have been included as supplementary material: (1) clinical outcomes excluding patients undergoing emergency TAVR for primary aortic regurgitation; (2) comparison of baseline demographic and clinical characteristics between patients receiving MCS pre-TAVR and patients receiving MCS intra-/post-TAVR.
