# Supplementary material for: Outcomes of Emergency Transcatheter Aortic Valve Replacement
Source: J Interv Cardiol. 2019 Nov 3;2019:7598581. doi: 10.1155/2019/7598581 (PMC6875395; doi:10.1155/2019/7598581)
Supplement: Supplementary Materials — Two additional tables have been included as supplementary material: (1) clinical outcomes excluding patients undergoing emergency TAVR for primary aortic regurgitation; (2) comparison of baseline demographic and clinical characteristics between patients receiving MCS pre-TAVR and patients receiving MCS intra-/post-TAVR. [file 7598581.f1.docx]

Supplemental Table 1: Outcomes excluding primary aortic regurgitation

| In-hospital outcomes |  |
| --- | --- |
| Intra-procedural mortality, n (%) | 1 (3.6) |
| In-hospital mortality, n (%) | 5 (17.9) |
| Procedural success, n (%) | 26 (92.9) |
| AR post procedure- none/mild, n (%) | 27 (100.0) |
| AR post procedure- moderate/severe, n (%) | 0 (0.0) |
| Stroke, n (%) | 3 (11.1) |
| Major bleed, n (%) | 2 (7.4) |
| Major vascular complication, n (%) | 5 (18.5) |
| Myocardial infarction, n (%) | 0 (0.0) |
| New PPM, n (%) | 2 (7.4) |
| Acute kidney injury stage III, n (%) | 4 (14.8) |
| Initiation of dialysis post TAVR, n (%) | 4 (14.8) |
| LOS [days], median (IQR) | 8 (5,14) |
|  |  |
| All-Cause Mortality |  |
| In-hospital, n (%) | 5 (17.9) |
| Total follow up, n (%) | 11 (39.2) |
|  |  |
| Cause of death |  |
| Cardiovascular mortality, n (%) | 6 (54.5) |
| Non-cardiovascular mortality, n (%) | 5 (45.4) |

AR: aortic regurgitation; PPM: permanent pacemaker; TAVR: transcatheter aortic valve replacement; LOS: length of stay

Supplemental Table 2: Comparison of baseline demographics and clinical characteristics by mechanical circulatory support pre and intra/post transcatheter aortic valve replacement

| **Demographics** | MCS pre-TAVR | MCS intra/post-TAVR |
| --- | --- | --- |
| Age [years], mean (SD) | 75.6 (15.2) | 70.2 (11.6) |
| Male gender, n (%) | 9 (90.0) | 6 (100.0) |
| BSA [kg/m^2^], mean (SD) | 1.99 (0.25) | 1.98 (0.20) |
| Race |  |  |
| White, n (%) | 7 (70.0) | 5 (83.3) |
| Black, n (%) | 1 (10.0) | 0 (0.0) |
| Hispanic, n (%) | 1 (10.0) | 0 (0.0) |
| Asian, n (%) | 0 (0.0) | 0 (0.0) |
| Other/unknown, n (%) | 1 (10.0) | 1 (16.7) |
| **History and Risk Factors** |  |  |
| Current/recent smoker, n (%) | 2 (20.0) | 2 (33.3) |
| Hypertension, n (%) | 8 (80.0) | 4 (66.7) |
| Diabetes mellitus, n (%) | 3 (30.0) | 3 (50.0) |
| Peripheral artery disease, n (%) | 2 (20.0) | 0 (0.0) |
| Atrial fibrillation, n (%) | 5 (50.0) | 5 (83.3) |
| Coronary artery disease, n (%) | 9 (90.0) | 3 (50.0) |
| Chronic kidney disease (GFR<60 ml/min; not on HD), n (%) | 4 (40.0) | 2 (33.3) |
| Chronic kidney disease on HD, n (%) | 1 (10.0) | 0 (0.0) |
| COPD, n (%) | 0 (0.0) | 2 (33.3) |
| Severe COPD, n (%) | 0 (0.0) | 1 (16.7) |
| Prior PCI, n (%) | 2 (20.0) | 1 (16.7) |
| Prior CABG, n (%) | 2 (20.0) | 1 (16.7) |
| Prior sternotomy, n (%) | 2 (20.0) | 1 (16.7) |
| Prior SAVR, n (%) | 2 (20.0) | 0 (0.0) |
| Prior stroke, n (%) | 0 (0.0) | 0 (0.0) |
| Immunocompromise, n (%) | 0 (0.0) | 1 (16.7) |
| Known history of CHF, n (%) | 9 (90.0) | 5 (83.3) |
| **Pre-Procedure Status** |  |  |
| Prior MI, n (%) | 2 (20.0) | 2 (33.3) |
| Cardiogenic shock within 24 hours, n (%) | 8 (80.0) | 5 (83.3) |
| ACS at hospital presentation, n (%) | 7 (70.0) | 4 (66.7) |
| ACS at time of TAVR, n (%) | 4 (40.0) | 4 (5.7) |
| Heart rate [beats/min], mean (SD) | 96.4 (23.5) | 99.0 (23.2) |
| BNP [pg/mL], mean (SD) | 2217.0 (1759.2) |  |
| Creatinine [mg/dL], mean (SD) | 1.56 (1.23) |  |
| Intubated, n (%) | 12 (38.7) |  |

Supplemental Table 2 (continued):

| **Echocardiographic Variables** | MCS pre-TAVR | MCS intra/post-TAVR |
| --- | --- | --- |
| Aortic valve area [cm^2^], mean (SD) | 0.78 (0.25) | 1.4 (1.29) |
| </=1.0 cm^2^, n (%) | 9 (90.0) | 4 (66.7) |
| Mean valve gradient [mmHg], mean (SD) | 31.2 (7.8) | 23.1 (13.3) |
| Moderate/severe AR, n (%) | 2 (20.0) | 2 (33.3) |
| Moderate, severe MR, n (%) | 2 (20.0) | 2 (33.3) |
| LVEF [%], mean (SD) | 27 (13.7) | 23.6 (11.9) |
| LVEF<35%, n (%) | 8 (80.0) | 5(83.3) |
| Stroke volume index [ml/m^2^], mean (SD) | 32.6 (15.8) | 26.6 (4.4) |
| </= 35 ml/m^2^, n (%) | 8 (80.0) | 5 (83.3) |
| Estimated PASP [mmHg], mean (SD) | 46.7 (8.2) | 50.8 (7.7) |
| **Hemodynamic Variables** |  |  |
| Right atrial pressure [mmHg], mean (SD) | 9.4 (3.0) | 17.5 (3.7) |
| *Pulmonary artery pressure* |  |  |
| Systolic [mmHg], mean (SD) | 49.2 (12.2) | 47.2 (7.8) |
| Diastolic [mmHg], mean (SD) | 23.7 (5.7) | 29 (6.5) |
| Mean [mmHg], mean (SD) | 32.2 (7.1) | 35.5 (6.5) |
| Pulmonary capillary wedge pressure or LVEDP [mmHg], mean (SD) | 18.3 (5.1) | 26.8 (5.8) |
| Mean arterial pressure [mmHg], mean (SD) | 66.6 (7.2) | 69.2 (8.1) |
| Cardiac output [l/min], mean (SD) | 5.2 (1.7) | 5.1 (3.2) |
| Cardiac index [l/min/m^2^], mean (SD) | 2.7 (1.0) | 2.5 (1.6) |
| Cardiac power index [W/m^2^], mean (SD) | 0.39 (0.13) | 0.39 (0.25) |

BSA: body surface area; GFR: glomerular filtration rate; HD: hemodialysis; COPD: chronic obstructive pulmonary disease; PCI: percutaneous coronary intervention; CABG: coronary artery bypass grafting; SAVR: surgical aortic valve replacement; CHF: congestive heart failure; MI: myocardial infarction; ACS: acute coronary syndrome; TAVR: transcatheter aortic valve replacement; BNP: B-type natriuretic peptide; AR: aortic regurgitation; MR: mitral regurgitation; LVEF: left ventricular ejection fraction; PASP: pulmonary artery systolic pressure; LVEDP: left ventricular end diastolic pressure; MCS: mechanical circulatory support
